# Moral injury in clinical and academic medicine—it is time to act

**DOI:** 10.1016/j.bjao.2025.100525

**Published:** 2026-01-20

**Authors:** Britta S. von Ungern-Sternberg, Aine Sommerfield, Karin Becke-Jakob

**Affiliations:** 1 Department of Anaesthesia and Pain Medicine, Perth Children’s Hospital, Nedlands, Western Australia, Australia; 2 Anaesthesia and Pain Management Program, Institute for Paediatric Perioperative Excellence, The University of Western Australia, Perth, Western Australia, Australia; 3 Department of Anaesthesia and Pain Medicine, Division of Emergency Medicine, Anaesthesia and Pain Medicine, The University of Western Australia, Perth, Western Australia, Australia; 4 Perioperative Medicine Team, The Kids Research Institute Australia, Nedlands, Western Australia, Australia; 5 Department of Anaesthesia, University Children’s Hospital Basel, Basel, Switzerland

**Keywords:** academic medicine, burnout, clinical ethics, clinical research, ethics, moral injury

## Abstract

When doctors working within healthcare systems under pressure perpetrate, witness, or fail to prevent acts that contradict their own moral or ethical values and expectations, it can lead to moral distress or moral injury. This can result from active behaviour and from purposeful inactive behaviour. It is a growing and critical concern, representing significant distress that extends far beyond traditional concepts such as burnout. This article discusses moral injury in clinical and academic medicine and actively gives suggestions to prevent and address moral injury.

A growing psychological toll is being experienced by doctors working within healthcare systems under pressure.^1^ The umbrella term ‘moral stressors’ encompasses phenomena such as moral distress and moral injury—concepts that have rapidly gained attention in recent years.^2^ In 2021, the British Medical Association surveyed all doctors on their experiences with these issues.^1^ Although just fewer than half had not heard of moral injury and moral distress previously, more than three-quarters reported that moral distress resonated with their work experiences, and more than half reported the same about moral injury.^1^ Interestingly, this phenomenon predates the COVID-19 pandemic but has been further exacerbated since then.^3^ Structural inadequacies, especially insufficient staffing and bureaucratic constraints, were the most cited causes. Disparities across ethnicity, seniority, and specialty further underline the systemic nature of the issue.

So, what is the problem? Defining and understanding these experiences is not merely an academic exercise but a crucial step toward safeguarding the well-being and integrity of the medical workforce. It is time to take a closer look at the issue of moral injury and strategies to prevent possible consequential damage.

## Definitions

The concept of moral distress and moral injury is not new. In 1984, Jameton^4^ defined moral distress as a ‘psychological disequilibrium in which a nurse feels distressed after recognising an ethically suitable action for his or her patient but being unable to take that action’. Moral injury was first defined by Shay,^5^ a psychologist working with veterans. He described the phenomenon of lifelong psychological injury that occurs when a person’s sense of ‘what is right’ is violated, and recovery is only possible if this violation is addressed.

Including findings on social cognitive theories of post-traumatic stress disorder (PTSD), Litz and colleagues^6^ defined moral injury as ‘the lasting psychological, biological, spiritual, behavioural and social impact of perpetrating, failing to prevent, or bearing witness to an act that transgresses deeply held moral beliefs and expectations’.

To put it simply, moral injury has been described as resulting from multiple single episodes of moral distress. This has been recently taken up by the definition published by the British Medical Association: ‘Moral distress refers to the psychological unease generated where professionals identify an ethically correct action to take but are constrained in their ability to take that action’ and ‘Moral injury arises where sustained moral distress leads to impaired function or longer-term psychological harm’.^1^

The most recent proposal for a definition of moral injury by VanderWeele and colleagues^7^ focusses on the moral content of the experience rather than the symptoms: ‘Persistent distress that arises from a personal experience that disrupts or threatens: (a) one’s sense of the goodness of oneself, of others, of institutions, or of what are understood to be higher powers, or (b) one’s beliefs or intuitions about right and wrong, or good and evil’.

It is important to note that moral injury is not universally experienced. It has been found to be more common in staff from ethnic minority backgrounds.^1^ A study among emergency medicine department chairs highlighted the different motivations of males and females; although male department chairs were motivated by the gain in influence, females were primarily motivated by the urge to make a difference for their patients and the system.^8^, ^9^, ^10^ Individuals driven by a motivation to help others may be more vulnerable to moral injury when this is not possible owing to systemic issues, such as resource constraints.

It is important to distinguish moral distress and moral injury from vicarious trauma (also known as secondary traumatic stress or compassion fatigue) and burnout.^11^ Burnout is traditionally framed as an individual problem. It suggests that the clinician is overwhelmed, not resilient enough, or needs to improve self-care. As a result, the ‘solutions’ are typically directed at the individual (e.g. mindfulness, wellness programs, coping strategies). Conversely, moral injury is fundamentally a system-based problem. It arises when clinicians are forced to act against their moral or professional values because of structural barriers such as unsafe staffing, time pressures, resource shortages, and conflicting policies. The responsibility for prevention or repair lies with the organisation, not the individual. Burnout frames distress as a failure of the clinician. Moral injury correctly frames it as a failure of the system.

Although moral injury is often confused with burnout, the preventative actions and treatments can differ significantly^12^, ^13^, ^14^; however, both require multilevel interventions that may overlap, including systematic workplace efficiency changes, a culture of well-being, and individual factors. Furthermore, moral injury often requires significant organisational and cultural changes^13^ to address systemic issues that may be linked to toxic leadership, and systems in which personal, sometimes hidden, agendas take priority over the team or the patients.^15^ The differences and overlaps of moral stressors are outlined in Table 1.

**Table 1:** Moral stressors: differences and overlaps among moral distress, moral injury, vicarious trauma, and burnout. PTSD, post-traumatic stress disorder.

	Definition	Typical causes	Impact	Key differences	Summary
Moral distress	Psychological discomfort when one knows the ethically right action but feels constrained from acting on it.	Institutional barriers, hierarchy, legal constraints, policies, lack of resources.	Erodes professional integrity, may lead to withdrawal or resignation.	More situational/episodic, linked to ethical dilemmas and systemic barriers.	‘I know the right thing but cannot do it’.
Moral injury	Lasting psychological/spiritual harm after committing, witnessing, or failing to prevent actions that transgress deeply held moral values.	Participating in or witnessing perceived wrongs (e.g. rationing care, neglect owing to systemic failure, unethical research conduct causing patient harm).	Long-term psychological harm, PTSD-like symptoms, erosion of meaning.	Deeper and more enduring than moral distress; associated with *violated moral identity*.	‘I did (or witnessed) something that betrayed my core values’.
Vicarious trauma	Emotional residue from repeated exposure to others’ trauma.	Continuous empathetic engagement with patients’ suffering, traumatic stories, emergency/end-of-life care.	Affects empathy, emotional resilience, professional functioning.	Originates from *absorbing others’ trauma*, not necessarily from systemic barriers.	‘I carry the weight of others’ suffering’.
Burnout	A state of emotional, physical, and mental exhaustion as a result of prolonged occupational stress.	Excessive workload, lack of support, understaffing, poor work–life balance.	Decreased quality of care, absenteeism, intention to leave profession.	Primarily stress-related, not necessarily moral/ethical in nature.	‘I am depleted and exhausted from chronic stress’.

### A few more theoretical details about morals and moral injury

Morals are deeply held beliefs about what constitutes good conduct and are influenced by upbringing, culture, and personal experiences. Morals guide our everyday decisions and actions. Moral injury is a betrayal of moral principles, an ethical insult, a threat to a person’s integrity by systemic or authoritative forces. It is commonly linked to systemic issues or (in)actions within a system that conflict with deeply held moral values of the person affected.^16^ Regardless of who is responsible for the action leading to moral injury, whether the person themselves committed it or only bore witness, moral injury disrupts the person’s identity of self and can lead to subsequent significant, destructive feelings.^17^ The core features of moral injury are reflected in symptoms such as intense negative emotions and erosion of the person’s professional identity, social isolation, emotional numbing, difficulty to self-forgive, and withdrawal. In some instances, moral injury can be linked to depression and PTSD.

## Clinical examples

For many clinicians working in resource-constrained or primarily profit-seeking healthcare systems, moral injury can occur as a result of inherent systemic limitations on their capacity to provide the best care for their patients, forcing them to act against their own moral standards of putting their patients’ needs first. In clinical practice, the challenge of knowing what care an individual patient requires, but the inability to provide this care owing to various reasons, can lead to moral injury.

There are numerous examples of moral conflicts that most of us have probably experienced: patient, team, system, leadership, and equity issues. First, how can we deal with the situation when we observe an inexperienced/inexpert surgeon having difficulty with a complex operation? Should the anaesthesiologist contact a more senior surgeon/the surgeon’s chief to come assist or avoid embarrassing the surgeon and undermining their ability while letting them continue to struggle (also preventing criticism of oneself)? Second, there might be challenges with obtaining truly informed consent from anxious patients, possibly with limited health literacy or language barriers, in a time-pressured environment, which may create moral distress when anaesthesiologists feel they are not adequately ensuring patient comprehension. Third, how can we cope with legitimate doubts in futile operations on patients who are likely to die? Fourth, what about anaesthesiologists participating in novel organ procurement techniques with minimal control of decision-making?^18^ Fifth, how to act when we notice that iatrogenic complications are played down deliberately? Finally, what about inadequate research conducted that might put patients at risk?

Clinicians are often drawn to medicine by a strong sense of purpose; the desire to help their patients, to teach, or to advance knowledge by research. This sense of purpose is often deeply anchored in a person’s values. In our specialty of anaesthesia and intensive care, we deal with a large number of complex patients, with critical illnesses, all within a multidisciplinary area, interacting with many different individuals with different agendas and their own internal and external pressures. This high-stakes, often fast-paced, decision-making environment is particularly vulnerable to moral injury, as time for team communication and ethical considerations may be limited in this setting. The constant double binds of endeavouring to take the best care of our patients, while considering the broader context of the hospital, the healthcare system, the general health economics, and our own productivity, just to name a few, can lead to moral injury.

Clinicians may witness colleagues or even leaders engaging in behaviours that are questionable or that violate ethical standards. They may bear witness to the detrimental effects of the system on their vulnerable patients, for example, restrictions on diagnosis or treatment options, owing to the level of insurance coverage a patient may have. Clinicians may feel powerless or unsafe about intervening or speaking up in these incidents. This may lead to clinicians experiencing a deep sense of having failed patients (even if not under their direct care) or themselves, even if the circumstances were beyond their own control.^19^ Moral injury in staff can have an indirect impact on patient care, through reduced empathy, an increased likelihood of medical errors, decreased staff engagement, and higher staff turnover.

Observing unethical behaviour by colleagues, superiors, or more generally by the system which is broader than just the organisation and can encompass entities such as governments, insurance companies, and the healthcare service itself, particularly when patients are being (potentially) harmed, can lead to significant moral injury, especially when the system fails to act and address the misconduct. When the fear of a powerful perpetrator is too great for an institution, or the fear of negative repercussions from potential media coverage after misconduct revelations at an institution is too large, silence or inactivity may follow.

## Academic considerations

What about academia? The pursuit of scientific advancement, the enthusiasm of the researchers, the one-sided perspective of some researchers focusing on innovation and improvements rather than holistic patient-centred care of the individual, the focus on personal gain and power, all have the potential to clash with immediate patient needs or ethical considerations, increasing the risk of harm to the patient and moral injury to other staff.

Clinician-scientists are typically highly driven individuals with strong moral compasses and a deep commitment to advancing science and patient care. They constantly have to weigh up ethical considerations during the conduct of their research. Ethical misconduct can have many facets: fabrication, falsification, plagiarism (FFP), negligence or incompetence, breaches in confidentiality, lack of informed consent, abuse, discrimination, etc. Witnessing ethical misconduct, or even worse, ethical misconduct where institutions quietly observe the misconduct without actively stopping it or sweeping it under the carpet, can lead to significant moral injury.

The blurring of the lines between clinical routine practice and research is particularly problematic, as patients, staff, and even members of the Ethics Committee may not fully understand the ‘cutting edge’, where standard clinical care ends and research begins. Ethics approval and informed consent may be especially challenging in vulnerable populations, including young children, particularly those with chronic, life limiting conditions, or the frail and older individuals.

Therapeutic misconception^20^ might entice patients to agree to additional (unnecessary) interventions or otherwise relatively riskier interventions without a complete understanding of the risk, need (or lack thereof), benefit, or their personal context.^21^ If the line between clinical care and research is not clear and visible to both participants and researchers at all times, then it can cause not only harm to the patient but also moral injury to healthcare professionals who witness this occurring.

Access to experimental treatments, which may be seen as beneficial or even the last hope, may be limited; inclusion or exclusion in studies can therefore lead to ethical dilemmas and a feeling of injustice for healthcare workers. The constant push for innovation, success, and being at the forefront can put significant pressure on researchers to achieve certain results and follow promising paths. In some institutions, this pressure to publish is even more pronounced, as some require publications for promotion, which in turn leads to higher standing, more influence, or higher income.

Although the majority of researchers behave ethically and in line with ethical guidelines, there is unfortunately also the potential for significant grey zones, leading to corners being cut, inaccurate data interpretation, selective reporting, or even fraud to reach their aim. This corner-cutting can be a source of moral injury. Ensuring that researchers do not work in these potential grey zones will reduce both ethical misconduct and potential moral injury. This necessitates working closely with a diverse range of patient representatives and consumers from the conceptualisation of any trial to the translation and implementation.^22^ We should ask colleagues outside our own institution to peer-review our projects, to improve research quality and avoid moral pitfalls.

## Systemic solutions

Moral injury is an issue that extends far beyond the healthcare system. Professional and collegial peer support networks can be helpful in this regard before institutional frameworks come into play.

When establishing systemic solutions, the following should be kept in mind: moral injury can occur when the healthcare professional feels compelled or forced to participate in questionable or unethical behaviour, clinically or academically, but also if they are solely a witness. When questionable or unethical practices become so common or remain unaddressed, they become normalised.^23^ This can erode the overall culture of the entire system and a person’s moral compass. Working for that particular system becomes increasingly difficult, and the person will start to question their own values and whether or not they must conform to these lower standards to survive and succeed.

Systemic solutions for moral injury must address the tension between institutional goals, such as economic efficiency and reputation management, and healthcare provider goals focused on patient care. This tension is particularly significant in both economically strained and profit-driven systems, where financial pressures can conflict with the ethical imperatives of healthcare providers. Nonspecific health programmes offered to staff to improve their well-being will not fix moral injury; rather, they require a healthcare environment based on psychological safety that truly values clinicians, gives them sufficient time to spend with their patients to listen, to gain each other’s trust, to understand, and be able to show true compassion. Putting the patient’s best interest first, above profit, institutions can include validated moral distress and moral injury assessments into their standard well-being assessments.^24^

So, what can we do to counteract moral injury in practical terms? Moral injury requires system-level changes (Table 2 details characteristics of morally centred organisations that may assist with preventing moral injury and distress); these changes need time. However, we cannot wait passively; each of us individually must play our role in promoting a safe and inclusive environment. We should reflect on our own behaviour, and support those who are negatively affected. We should actively speak up against ethical misconduct and complicit, inactive, or toxic leadership.^15^^,^^25^ Institutions must protect whistleblowers, give them safe, anonymous pathways for speaking up,^25^ and implement robust complaint investigation systems with real consequences for misconduct. A lack of consequences for misconduct can not only lead to a contamination of the scientific record and potential harm to patients and public health but also lead to a discouragement of whistleblowers. A culture of impunity fosters fear, silence, and continued unethical practice, further deepening the moral injury.

**Table 2:** Characteristics of morally centred organisations which may assist with preventing moral injury and distress.

System-level
• Sufficient staffing to counteract workload pressures
• Supportive leadership with a culture of transparency, honest and open communication
• Implement programmes to identify and address moral injury early
• Empower clinicians to have more agency in patient care decisions
• Reduce influence of administrator-driven clinical medicine
• Reduce administrative burdens and bureaucracy
• Prioritise patient and staff health over profit
• Promote genuine interest in feedback from clinicians
• Learn from mistakes and near misses
• Maintain sound ethics and governance processes, clinically and academically
• Take appropriate actions against ethical misconduct, clinically and academically
• Take appropriate actions against racism, disability bias, homophobia/transphobia, sexual harassment, or other discriminatory behaviour
• Promote a positive, diverse culture
• Create an environment where it is safe to speak up
• Support open disclosure of medical errors, unethical or toxic behaviour

**Actions on a departmental or individual**

• Create safe spaces for staff to speak about their experiences and moral injury without judgement
• Develop peer support networks
• Speak out against harmful policies or practices (if safe to do so)
• Promote open communication to policymakers and administrators about the realities of health care and the real-life impacts of systemic constraints on patients and staff
• Open disclosure

The strategies detailed above are systematic preventative strategies rather than interventions for healthcare workers already suffering from moral distress and moral injury, which are often harder to access. Examples of moral distress/injury interventions with supportive evidence include Acceptance and Commitment Therapy for Moral Injury (ACT-MI), Adaptive Disclosure, and Trauma-Informed Guilt Reduction.^26^

In summary, anaesthesiologists should recognise that the emotional strain stemming from moral injury—its profound impact on one’s sense of integrity—can be a major driver of frustration, exhaustion, detachment, diminished professional fulfilment, and growing disillusionment in clinical practice, beyond what is typically described as burnout.

The path forward requires courageous leadership, systemic reform, and a renewed commitment to the foundational ethical principles of medicine. The imperative is to protect the moral compass of healthcare professionals by addressing the systemic failings that compromise their ability to provide compassionate, ethical care.

We should endeavour to recognise moral injury in ourselves and others, and support institutional efforts to address it. A key factor in addressing moral distress is to help move people and communities from helpless inaction to active agency. If we all work together, we can make a difference; together, we can improve not only our culture but also our own well-being and, at the same time, the care we give our patients.

## Funding

Stan Perron Charitable Foundation (to BSvUS) and National Health and Medical Research Council Investigator Grant (2009322; to BSvUS).

## Declaration of interests

The authors declare that they have no conflicts of interest.
